# Statins in the Treatment of Chronic Heart Failure: A Systematic Review

**DOI:** 10.1371/journal.pmed.0030333

**Published:** 2006-08-22

**Authors:** Pim van der Harst, Adriaan A Voors, Wiek H van Gilst, Michael Böhm, Dirk J van Veldhuisen

**Affiliations:** 1 Department of Cardiology, Thoraxcenter, University Medical Center Groningen, University of Groningen, Netherlands; 2 Department of Clinical Pharmacology, University Medical Center Groningen, Groningen, Netherlands; 3 Klinik and Poliklinik Innere Medizin III, Universität des Saarlandes, Homburg, Germany; Glasgow Royal Infirmary, United Kingdom

## Abstract

**Background:**

The efficacy of statin therapy in patients with established chronic heart failure (CHF) is a subject of much debate.

**Methods and Findings:**

We conducted three systematic literature searches to assess the evidence supporting the prescription of statins in CHF. First, we investigated the participation of CHF patients in randomized placebo-controlled clinical trials designed to evaluate the efficacy of statins in reducing major cardiovascular events and mortality. Second, we assessed the association between serum cholesterol and outcome in CHF. Finally, we evaluated the ability of statin treatment to modify surrogate endpoint parameters in CHF.

Using validated search strategies, we systematically searched PubMed for our three queries. In addition, we searched the reference lists from eligible studies, used the “see related articles” feature for key publications in PubMed, consulted the Cochrane Library, and searched the ISI Web of Knowledge for papers citing key publications.

Search 1 resulted in the retrieval of 47 placebo-controlled clinical statin trials involving more than 100,000 patients. CHF patients had, however, been systematically excluded from these trials. Search 2 resulted in the retrieval of eight studies assessing the relationship between cholesterol levels and outcome in CHF patients. Lower serum cholesterol was consistently associated with increased mortality. Search 3 resulted in the retrieval of 18 studies on the efficacy of statin treatment in CHF. On the whole, these studies reported favorable outcomes for almost all surrogate endpoints.

**Conclusions:**

Since CHF patients have been systematically excluded from randomized, controlled clinical cholesterol-lowering trials, the effect of statin therapy in these patients remains to be established. Currently, two large, randomized, placebo-controlled statin trials are under way to evaluate the efficacy of statin treatment in terms of reducing clinical endpoints in CHF patients in particular.

## Abstract 2

A systematic review found that patients with heart failure have been excluded from randomised controlled trials on the use of statins. Evidence from other studies on the effectiveness of statins for patients with heart failure is weak and conflicting.

## Editors' Summary

**Background.**

When medical researchers test a drug—or some other treatment—for a particular medical condition, they often decide not to include in their study anyone who has, in addition to the disease they are interested in, certain other health problems. This is because including patients with two or more conditions can complicate the analysis of the results and make it hard to reach firm conclusions. However, excluding patients in this way can result in uncertainty as to whether treatments are effective for *anyone* who suffers from the disease in question, or just for people like those who took part in the research.

A great deal of research has been conducted with drugs known as statins, which lower cholesterol levels in the blood. (A raised level of cholesterol is known to be a major risk factor for cardiovascular disease, which causes heart attacks and strokes.) As a result of this research, statins have been accepted as effective and safe. They are now, in consequence, among the most commonly prescribed medicines. Heart failure, however, is not the same thing as a heart attack. It is the name given to the condition where the muscles of the heart have become weakened, most often as a result of aging, and the heart becomes gradually less efficient at pumping blood around the body. (Some people with heart failure live for many years, but 70% of those with the condition die within ten years.) It is common for people with cardiovascular disease also to have heart failure. Nevertheless, some researchers who have studied the effects of statins have made the decision not to include in their studies any patients with cardiovascular disease who, in addition, have heart failure.

**Why Was This Study Done?**

The researchers in this study were aware that patients with heart failure have often been excluded from statin trials. They felt it was important to assess the available evidence supporting the prescription of statins for such patients. Specifically, they wanted to find out the following: how often have patients with heart failure been included in statin trials, what evidence is available as to whether it is beneficial for patients with heart failure to have low cholesterol, and what evidence is there that prescribing statins helps these patients?

**What Did the Researchers Do and Find?**

They did not do any new work involving patients. Instead, they did a very thorough search for all relevant studies of good quality that had already been published and they reviewed the results. “Randomized clinical trials” (RCTs) are the most reliable type of medical research. The researchers found there had been 47 such trials (involving over 100,000 patients) on the use of statins for treating cardiovascular disease, but all these trials had excluded heart failure patients. They found eight studies (which were not RCTs) looking at cholesterol levels and heart failure. These studies found, perhaps surprisingly, that death rates were higher in those patients with heart failure who had low cholesterol. However, they also found 18 studies (again not RCTs) on the use of statins in patients with heart failure. These 18 studies seemed to suggest that statins were of benefit to the patients who received them.

**What Do These Findings Mean?**

The evidence for or against prescribing statins for people with heart failure is limited, conflicting, and unclear. Further research involving RTCs is necessary. (Two such trials are known to be in progress.)

**Additional Information.**

Please access these Web sites via the online version of this summary at http://dx.doi.org/10.1371/journal.pmed.0030333.

- General information about statins is available from the Web site of Patient UK
- The American Heart Association Web site is a good source of information about all types of heart disease, including heart attacks and heart failure
- For a definition of randomized controlled trials see Wikipedia, a free online encyclopedia that anyone can edit
- More detailed information about the quality of evidence from medical research may be found in the James Lind Library

## Introduction

The efficacy of 3-hydroxy-3-methylglutaryl coenzyme-A reductase inhibitors, or statins, in reducing morbidity and mortality in patients with documented coronary artery disease (CAD), or at risk of developing it, has been overwhelmingly and indisputably proven during the past decade [1–15]. However, it is unclear whether statin treatment is also beneficial in patients with established chronic heart failure (CHF) [16,17]. To investigate the strength of the evidence supporting statin treatment in patients with CHF, three systematic literature searches were carried out. The objectives of these searches were as follows: (i) to assess the participation of CHF patients in randomized, placebo-controlled clinical trials designed to evaluate the efficacy of statin treatment in reducing major cardiovascular events and death, (ii) to determine the relationship between cholesterol levels and outcome in patients with established CHF, and (iii) to assess the reported efficacy parameters and results of statin treatment in patients with established CHF.

## Methods

This study and reporting was performed adhering to the QUOROM [18] statement when possible (see Protocol S1).

### Literature Search

The search to identify all potentially relevant studies was initiated using search tools provided by PubMed (http://www.ncbi.nlm.nih.gov/entrez/query/static/clinical.shtml; used July 2005). These search tools have recently been validated by Haynes et al. as optimizing retrieval [19]. The scope filters employed were all set to “broad, sensitive search” as recommended for research applications. We included papers published in all languages. We constructed one query per objective (see Protocol S2). In addition, we consulted with experts, searched our own files, reviewed reference lists from eligible studies, used the “see related articles” feature for key publications in PubMed, consulted the Cochrane Library, and searched the ISI Web of Knowledge (http://scientific.thomson.com/webofknowledge) for publications that cited key publications.

### CHF Patients in Clinical Statin Trials

The first objective was to discover whether CHF patients were included in randomized clinical trials in which the efficacy of statin therapy in reducing major adverse cardiac events or death was either a primary or secondary endpoint. All randomized, placebo-controlled clinical trials, including those still ongoing, were included. Exclusion criteria relating to CHF (New York Heart Association [NYHA] class and left ventricular ejection fraction [LVEF]) were recorded.

### Cholesterol and CHF

The second objective was to assess the association between cholesterol levels and outcome in patients with established CHF. We anticipated retrieving predominantly small-scale, post hoc observational studies using various methodologies and analyzing different cholesterol cutoff values. For this reason we did not assess the magnitude of the effect of cholesterol levels on CHF prognosis.

### Statin Treatment in CHF

The third objective was to assess the clinical evidence established in patients with CHF. All studies that aimed to evaluate statin treatment specifically in patients with established CHF were included. All reported efficacy parameters and study designs were assessed to estimate the strength of the clinical evidence. Case reports and serial case reports were excluded. Studies that investigated the effect of statins on the incidence of CHF in non-CHF populations were also excluded.

### Methods of Analysis

The results of the data extraction and assessment were summarized in structured tables.

## Results

### CHF Patients in Statin Trials

**Figure 1:**
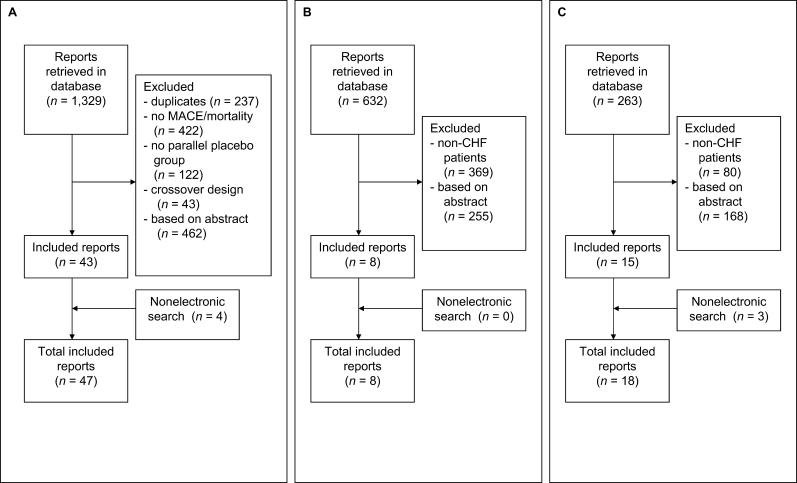
Flow Diagrams of Study Selection (A) Selection of studies about the handling of CHF patients in placebo-controlled statin trials. (B) Selection of studies about the association between cholesterol levels and mortality in CHF patients. (C) Selection of studies containing original data on statin treatment in patients with established CHF.

The electronic search retrieved 1,329 possibly relevant papers about CHF patients in statin trials. Of these, 47 randomized, controlled trials (including six ongoing) published between 1990 and 2005 fulfilled the eligibility criteria. Details of those papers excluded are shown in Figure 1A. In the 41 completed trials, a total of 106,167 patients were randomized to statin treatment or placebo. There were 14 primary prevention trials, 25 secondary prevention trials, and two trials that combined primary and secondary prevention (Table 1). Eighteen trials, with a total of 48,623 patients (46% of the total for all 41 trials), excluded all CHF patients. One trial excluded patients with LVEF less than 40% (*n =* 695, 0.7%), three trials excluded patients with LVEF less than 30% (*n =* 2,970; 3%), and one excluded patients with LVEF less than 35% (*n =* 460; <0.01%). Four trials excluded patients with NHYA III or higher (*n =* 10,855; 10%), four excluded patients with “severe” heart failure (21,327; 20%), and two trials excluded patients with symptomatic heart failure (*n =* 13,173; 12%). Eight papers provided no information in either the methodology or the baseline characteristics on whether CHF patients had been included (*n =* 8,064; 8%). The Cholesterol and Recurrent Events (CARE) trial excluded symptomatic heart failure, but reported a subgroup analysis of 706 patients with decreased LVEF (26%–40%). Statins were equally effective in patients with and without decreased LVEF [4]. Of the six ongoing trials, two are specifically designed to include CHF patients and will be discussed below [20,21].

**Table 1:**
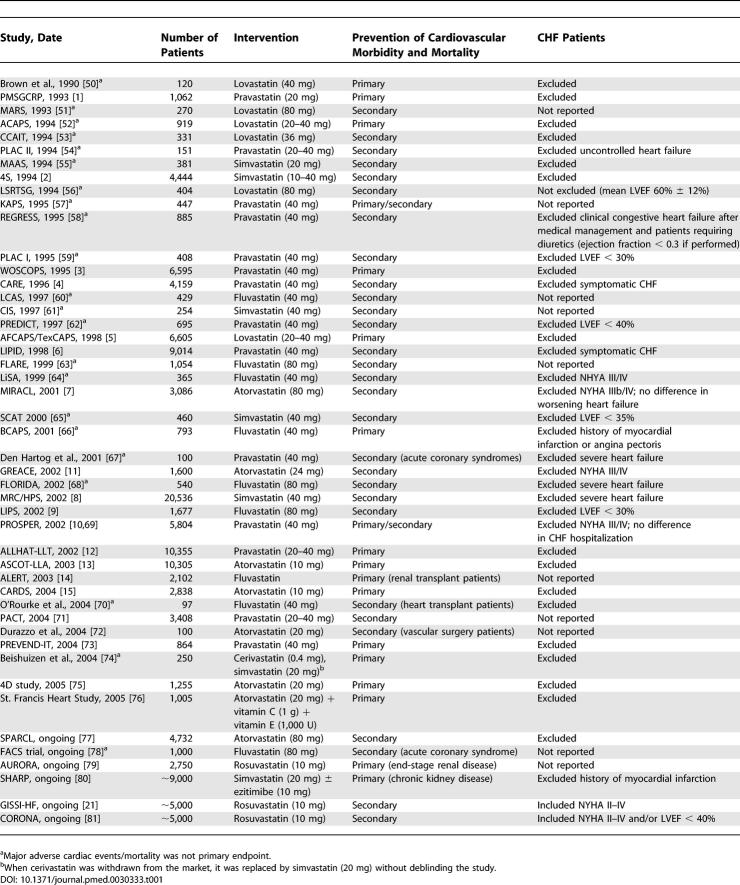
Placebo-Controlled Statin Trials and CHF Patients

### Cholesterol and CHF

The electronic search retrieved 632 possibly relevant papers about cholesterol and CHF. Eight papers describing ten independent patient populations (*n =* 3,879 in total) and published between 1998 and 2005 fulfilled the eligibility criteria (Figure 1B). Seven CHF populations were studied retrospectively (*n =* 2,837; 73%) and three populations were studied prospectively (*n =* 1,039; 27%). Both ischemic and non-ischemic cardiomyopathies were studied, and mortality was the outcome parameter in all but one study (*n =* 58; 1%). In 1998, Vredevoe et al. were the first to report that lower total cholesterol was associated with increased mortality in patients with advanced, idiopathic CHF [22]. Two studies sought to identify the optimum cholesterol cutoff value to predict mortality. In the study carried out by Horwich et al., which used “receiver operating characteristic” curves analysis, the optimum cutoff for total cholesterol in predicting mortality in CHF patients was 4.9 mmol/l (190 mg/dl), with a sensitivity of 70% for predicting mortality at 5 y [23]. Similarly, Rauchhaus et al. reported that the optimum cutoff value was 5.2 mmol/l (200 mg/dl) for predicting mortality at both 1 y (sensitivity 80%, specificity 63%) and 3 y (sensitivity 62%, specificity 74%). In this study, the chance of survival increased by approximately 25% for each millimoles/liter increment in total cholesterol (relative risk 0.75 [95% confidence interval (CI) 0.63–0.90]) [24]. On the other hand, a recent article by Christ et al. questioned the prognostic value of total cholesterol in patients with idiopathic dilated cardiomyopathy. Univariately, decreased total cholesterol was only a moderate predictor, and lost its significance after adjustment for increased left ventricular end-diastolic diameter, reduced LVEF, and increased NHYA class for the combined endpoint of death or heart transplantation (*p =* 0.34), and was borderline significant for the endpoint of death (*p =* 0.07; Table 2) [25]. Overall, results from eight patient populations and involving a total of 3,341 patients (86%) reported low cholesterol to be an independent predictor associated with mortality. Since methodology and categorization varied considerably among the reported populations, pooling of the results to determine the magnitude (hazard ratio [HR]) was inappropriate.

**Table 2:**
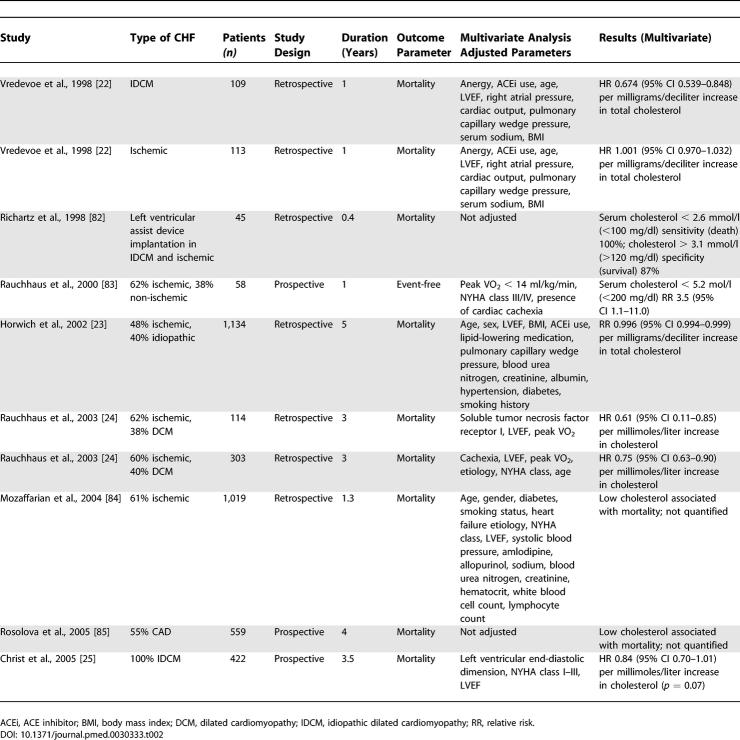
Cholesterol and CHF

### Statin Treatment in CHF

The electronic search retrieved 263 papers about statin treatment in CHF. Eighteen papers reporting efficacy parameters for statin treatment in CHF were included (Figure 1C). Outcome parameters differed considerably (Table 3) and did not allow pooling of data. Only one study, involving 24 patients, reported a reduction of coenzyme Q_10_, which is a possible adverse outcome [26]. All other studies reported favorable outcomes for almost all surrogate endpoint parameters or post hoc analyses of major adverse cardiac events or mortality (Table 3). Three studies had a prospective, randomized, placebo-controlled design involving a total of 104 patients. All these studies reported surrogate endpoints, and none had a single prespecified primary outcome parameter. One of the most noteworthy prospective studies was performed in idiopathic dilated cardiomyopathy [27]. Fifty-one patients were randomly assigned to simvastatin (up to 10 mg/d) or placebo. Using M-mode echocardiography with 2-D monitoring before and after 14 wk of treatment, Node et al. demonstrated improvement in functional capacity in statin-treated patients. In the statin group, 39.1% of patients had an improved functional class and 4.3% deteriorated. In contrast, in the placebo group 16% of patients improved and 12% deteriorated (*p* < 0.01). Another prospective, double-blind study randomized patients with non-ischemic dilated cardiomyopathy to cerivastatin (0.4 mg/d) or placebo [28]. Quality of life and exercise capacity increased significantly in the statin-treated patients. In addition, there was a trend towards increased LVEF and improved endothelial function. Recently, Landmesser et al. demonstrated improvement of endothelial function in CHF patients randomized to simvastatin (10 mg/d), but not in patients randomized to ezetimibe (10 mg/d) [29]. This suggests that improvement of endothelial function is independent of low-density lipoprotein cholesterol reduction.

**Table 3:**
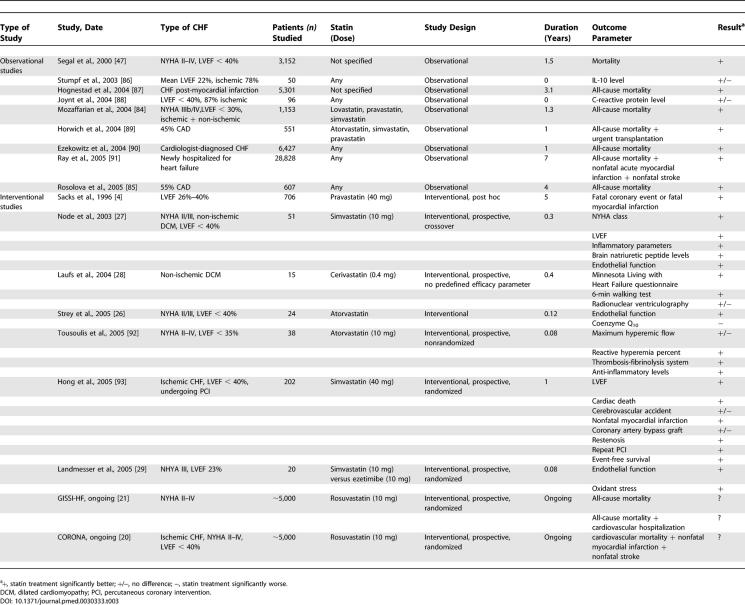
Observational and Interventional Studies Investigating Efficacy of Statin Treatment in CHF

Two large randomized, placebo-controlled clinical trials are currently under way, together involving in excess of 10,000 patients [20,21]. The Controlled Rosuvastatin multinational Study in Heart Failure (CORONA) will enroll about 4,950 patients with chronic, symptomatic systolic heart failure due to CAD [20]. The primary outcome is the composite endpoint of cardiovascular death or nonfatal myocardial infarction or nonfatal stroke. The Gruppo Italiano per lo Studio della Sopravvivenza nell'Infarto Miocardico (GISSI) heart failure trial will enroll approximately 7,000 patients to be randomized to n-3 polyunsaturated fatty acids or matching placebo, and if there is no clear indication for cholesterol-lowering therapy, patients will be further randomized to receive rosuvastatin or matching placebo [21]. The GISSI heart failure trial has two co-primary endpoints, namely all-cause mortality and the combined endpoint of all-cause mortality or cardiovascular hospitalizations. These two trials will formally assess statin treatment in CHF.

## Discussion

Although more than 100,000 patients have been studied in clinical trials evaluating the efficacy of statin treatment compared to placebo in primary and secondary prevention of cardiovascular morbidity and mortality, this investigation has revealed that the vast majority of published statin trials excluded patients with LVEF below 40% or NYHA III/IV. In addition, we report that in CHF patients, instead of high-serum cholesterol levels, low-serum cholesterol levels have been consistently associated with increased mortality rates. Finally, this study has revealed that although most studies assessing the effect of statins in CHF are encouraging, they only report surrogate efficacy parameters or are based on secondary analyses.

### Theoretical Considerations for Statin Treatment in CHF Patients

We conclude that statin treatment in CHF has never been assessed in a large clinical trial setting, which is the gold standard for clinical evidence (Table 1). In view of this, the question is to what extent can the results of studies performed in other patient groups be extrapolated to CHF populations?

There are several theoretical considerations that argue in favor or against statin treatment in CHF. The most relevant arguments against statin therapy are the endotoxin lipoprotein hypothesis, the coenzyme Q_10_ (ubiquinone) hypothesis, and the selenoprotein hypothesis. The endotoxin lipoprotein hypothesis is related to lower cholesterol levels and will be discussed below. The ubiquinone hypothesis reasons that the inhibition of mevalonate synthesis by statins decreases the production of ubiquinone (coenzyme Q_10_). Ubiquinone is involved in the production of ATP and therefore in meeting the metabolic demands of cells [30]. Another fundamental characteristic of ubiquinone is its antioxidant (free-radical-scavenging) properties. The selenoprotein hypothesis postulates that statins interfere with the enzymatic isopentenylation of selenocysteine tRNA and prevent its maturation to a functional tRNA molecule, resulting in a decrease in available selenoproteins [31]. Individuals with statin-induced myopathy have clinical and pathological features similar to those of syndromes associated with severe selenoprotein deficiency [31].

The potential beneficial effects of statin treatment in CHF include improved vascular function, improved neurohormonal status, and decreased development of left ventricular hypertrophy. Statin treatment has been shown to favorably affect endothelial function [29], as well as increasing capillary density [32] and circulating endothelial progenitor cells [33], and slowing the progression of coronary atherosclerosis [34]. In addition, statins also modify the major neurohormonal systems involved in CHF, decreasing responsiveness to angiotensin II, for example [35]. Statins also inhibit the beta-adrenergic receptor activation of Rac1 and consequently apoptosis [36]. Finally, by blocking the synthesis of mevalonate, statins reduce the development of left ventricular hypertrophy [37].

### Cholesterol in CHF

In middle-aged persons, hypercholesterolemia is a well-known risk factor predicting the development of CAD and long-term all-cause mortality. This association is, however, controversial in elderly patients and those with a wide range of chronic and acute diseases [38–40]. In the Framingham study, dyslipidemia initially appeared to be a risk factor for the development of CHF [41]. However, in a more detailed analysis of the same Framingham database, the association between total cholesterol and all-cause mortality was found to be positive at 40 y, negligible at 50–70 y, and negative at age 80 y and above [42]. In this context, it is important to note that the incidence and prevalence of CHF rises steeply with age, and that almost 80% of CHF patients are over 65 y of age. Despite these observations, however, the evidence from clinical trials proves that statin treatment is effective in elderly patients [43]. The Pravastatin in Elderly Individuals at Risk of Vascular Disease (PROSPER) trial extended the clinical evidence supporting statin treatment to elderly patients (>70 y) at high risk of developing cardiovascular disease and stroke [10].

Several studies have addressed the relation and relevance of serum cholesterol levels to outcome in CHF patients in particular, and the results consistently suggest that lower cholesterol is associated with increased mortality (Table 2). More specifically, for each millimoles/liter decrease in total cholesterol, mortality increases by 25% [23,24]. This phenomenon of “reverse epidemiology” in CHF is not unique for cholesterol levels, and also exists for body mass index and blood pressure [44]. Nevertheless, the above-mentioned studies are observational studies and therefore of limited value. Although most studies are corrected for several indicators, such as nutritional status and cachexia, they may have been inadequately adjusted for other confounders. Lower cholesterol may mark an end-stage disease epiphenomenon, due to reduced hepatic cholesterol synthetic capacity. The endotoxin lipoprotein hypothesis, however, offers a plausible explanation for the observed relationship. This hypothesis postulates that higher levels of cholesterol might be beneficial in CHF because of the ability of cholesterol to modulate inflammatory immune function [45]. CHF patients have increased serum cytokine levels, which might be linked to increased endotoxin levels [46]. Circulating cholesterol- and triglyceride-rich lipoproteins are natural nonspecific buffers of endotoxins. They have the capacity to bind and detoxify bacterial lipopolysaccharides. In parallel with raised lipopolysaccharide plasma concentrations, patients with edematous and severe CHF show substantial immune activation [46]. Episodes of endotoxemia may occur, and reducing lipoproteins could adversely affect lipopolysaccharide bioactivity modification [45].

### Clinical Evidence Supporting Statin Therapy in CHF

Several post hoc subgroup analyses of data from large clinical statin trials have been published, and have examined the effects of statins in CHF or on the development of CHF [2,4,10,47,48]. One recent study, the Treating to New Targets (TNT) study involving 10,001 CAD patients, investigated the efficacy of 80 mg versus 10 mg of atorvastatin (no placebo arm). In this study, high-dose statin therapy was associated with a 26% decrease in hospitalization for congestive heart failure, which was a predefined secondary efficacy outcome parameter (HR 0.74 [95%CI 0.59–0.96], *p =* 0.01) [49].

Statin therapy has been associated with reduced mortality in observational studies (Table 3). Nevertheless, the effects of statin use in CHF currently reported in the literature do not prove causality, and are susceptible to considerable confounders and biases. First, the single largest confounder in nonrandomized studies is probably the patient characteristics that are related to the physicians' decision to prescribe a statin. Second, some of these CHF studies were conducted at a time when beta-blockers and spironolactone were not generally used in severe heart failure. Third, most of the patients receiving statin treatment at discharge were on statin treatment before inclusion in the study, which further complicates the analyses. Finally, although statin therapy seems to reduce new onset of heart failure, this reduction could be related to effects on reduction of recurrent myocardial infarction and subsequent CHF, rather than the development of CHF without recurrent infarctions.

There are limited data on the effects of statin treatment in patients with established CHF (Table 3). At present, the available CHF trials appear encouraging, however, and their results warrant confirmation in large clinical trials.

### Strengths and Limitations

This paper is the first, to our knowledge, to systematically review the available data on the use of statin treatment in CHF patients. Limitations of the current study include the potential publication biases of the retrieved studies. Observational studies and post hoc analyses of clinical trials investigating the correlation between cholesterol levels and outcome in patients with CHF are exploratory in nature and may be more likely to be published if an association is found. Likewise, the published small-scale prospective studies evaluating statins specifically in CHF patients might also have suffered from publication bias since negative studies might have been considered underpowered.

### Conclusions

Despite widespread clinical use of statins for hypercholesterolemia and prevention of CAD, there is a paucity of data on the effects of statins on clinical outcome in CHF. Currently, the available experimental, post hoc, and observational data and theoretical considerations are conflicting. On the one hand, three lines of evidence point towards statins having a harmful effect in CHF. First, lower cholesterol levels have repeatedly been related to a poorer outcome in CHF patients. This may be related to the function of cholesterol as a scavenger for cardiodepressive and harmful endotoxins. Second, statins in CHF may adversely affect mitochondrial function through inhibition of ubiquinone. Third, statins may decrease selenoproteins, which could result in decreased myocardial function. On the other hand, evidence of beneficial effects of statin treatment in CHF is accumulating. First, beneficial effects of statins demonstrated in non-CHF conditions, e.g., on vascular function, atherosclerosis, and left ventricular hypertrophy, might also be beneficial in CHF. Second, clinical knowledge regarding statin treatment in CHF is generally favorable, although it is primarily based on retrospective post hoc studies performed within the scope of large clinical trials and on small prospective studies with surrogate endpoints.

Statin treatment in established CHF has never been formally assessed in a large clinical trial, the gold standard for clinical evidence. Therefore, there are currently no reliable clinical data on the efficacy of statins in CHF. Two large clinical trials are, however, now under way to clarify the effects of statin treatment in CHF [20,21]. These two studies will provide a more definite answer to whether or not we should initiate statin treatment in patients with established CHF.

## Supporting Information

Protocol S1 QUOROM Checklist (45 KB DOC)

Protocol S2 Search Queries Appendix (33 KB DOC)

## Abbreviations

CAD: coronary artery disease
CHF: chronic heart failure
CI: confidence interval
HR: hazard ratio
LVEF: left ventricular ejection fraction
NYHA: New York Heart Association
