# Supplementary material for: Statins in the Treatment of Chronic Heart Failure: A Systematic Review
Source: PLoS Med. 2006 Aug 22;3(8):e333. doi: 10.1371/journal.pmed.0030333 (PMC1551909; doi:10.1371/journal.pmed.0030333)
Supplement: Supplementary file 1 [file pmed.0030333.sd001.doc]

| Heading | Subheading | Descriptor | Reported? Y/N | Page number |
| --- | --- | --- | --- | --- |
| Title |  |  |  |  |
| Abstract |  | Use a structured format | Y | 2 |
|  | Objectives | The clinical question explicitly | Y | 2 |
|  | Data sources | The databases (ie, list) and other information sources | Y | 2 |
|  | Review methods | The selection criteria; methods for validity assessment, data abstraction, and study characteristics, and quantitative data synthesis in sufficient detail to permit replication | Y | 2 |
|  | Results | Characteristics of RCTs | Y | 2 |
|  | Conclusions | The main results | Y | 2 |
|  |  |  |  |  |
| Introduction |  | The explicit clinical problem, biological rationale for the intervention, and rationale for review | Y | 3 |
|  |  |  |  |  |
| Methods | Searching | The information sources, in detail, and any restrictions (years considered, publication status, etc) | Y | 3-4 |
|  | Selection | The inclusion and exclusion criteria (defining population, intervention, principal outcome, and study design) | Y | 5 |
|  | Validity assessment | The criteria and process used | Y | 5 |
|  | Data abstraction | The process of processes used | Y | 6 |
|  | Study characteristics | The type of study design, participants’ characteristics, details of intervention, outcome definition, and how clinical heterogeneity was assessed | Y | 5 |
|  | Quantitative data synthesis |  | Not applicable |  |
|  |  |  |  |  |
| Results | Trial flow | Provide a meta-analysis profile summarising trial flow | Y | Figure 1 |
|  | Study characteristics | Present descriptive data for each trial | Y | Table 1,2,3 |
|  | Quantitative data synthesis | Report agreement of the selection and validity assessment; present simple summary results; present data needed to calculate effect size and confidence … | Not applicable |  |
|  |  |  |  |  |
| Discussion |  | Summarise key findings; discuss clinical inferences based on internal and external validity; interpret the results in light of the totality of available evidence; describe potential biases in the review process; and suggest a future research agenda | Y | (9-)13 |
