# Supplementary material for: Statins in the Treatment of Chronic Heart Failure: A Systematic Review
Source: PLoS Med. 2006 Aug 22;3(8):e333. doi: 10.1371/journal.pmed.0030333 (PMC1551909; doi:10.1371/journal.pmed.0030333)
Supplement: Supplementary file 2 [file pmed.0030333.sd002.doc]

**Apendix**

*Objective*: How were patients with chronic heart failure (CHF) handled in placebo controlled statin trials?

**Query 1**

((placebo OR placebos OR "Placebos"[MeSH]) AND (statin OR statins OR HMG OR pitavastatin OR rosuvastatin OR atorvastatin OR simvastatin OR pravastatin OR lovastatin OR fluvastatin OR cerivastatin OR HMG)) AND ((clinical[Title/Abstract] AND trial[Title/Abstract]) OR clinical trials[MeSH Terms] OR clinical trial[Publication Type] OR random*[Title/Abstract] OR random allocation[MeSH Terms] OR therapeutic use[MeSH Subheading])

*Objective*: What is the reported association between cholesterol levels and motality in established CHF?

**Query 2**

((cholesterol AND ("Heart Failure, Congestive"[MeSH] OR heart failure)) AND ((clinical[Title/Abstract] AND trial[Title/Abstract]) OR clinical trials[MeSH Terms] OR clinical trial[Publication Type] OR random*[Title/Abstract] OR random allocation[MeSH Terms] OR therapeutic use[MeSH Subheading])) OR ((cholesterol AND ("Heart Failure, Congestive"[MeSH] OR heart failure)) AND (incidence[MeSH:noexp] OR mortality[MeSH Terms] OR follow up studies[MeSH:noexp] OR prognos*[Text Word] OR predict*[Text Word] OR course*[Text Word]))

*Objective*: Which original data has been published on statin treatment in etstablished CHF?

# Query 3

(("Heart Failure, Congestive"[MeSH] OR heart failure) AND (statin OR statins OR HMG OR pitavastatin OR rosuvastatin OR atorvastatin OR simvastatin OR pravastatin OR lovastatin OR fluvastatin OR cerivastatin OR HMG)) AND ((clinical[Title/Abstract] AND trial[Title/Abstract]) OR clinical trials[MeSH Terms] OR clinical trial[Publication Type] OR random*[Title/Abstract] OR random allocation[MeSH Terms] OR therapeutic use[MeSH Subheading])
